# 65% of Americans believe they are above average in intelligence: Results of two nationally representative surveys

**DOI:** 10.1371/journal.pone.0200103

**Published:** 2018-07-03

**Authors:** Patrick R. Heck, Daniel J. Simons, Christopher F. Chabris

**Affiliations:** 1 Autism & Developmental Medicine Institute, Geisinger Health System, Lewisburg, PA, United States of America; 2 Department of Psychology, University of Illinois, Champaign, IL, United States of America; 3 Institute for Advanced Study in Toulouse, Toulouse, France; Maastricht University, NETHERLANDS

## Abstract

Psychologists often note that most people think they are above average in intelligence. We sought robust, contemporary evidence for this “smarter than average” effect by asking Americans in two independent samples (total *N* = 2,821) whether they agreed with the statement, “I am more intelligent than the average person.” After weighting each sample to match the demographics of U.S. census data, we found that 65% of Americans believe they are smarter than average, with men more likely to agree than women. However, overconfident beliefs about one’s intelligence are not always unrealistic: more educated people were more likely to think their intelligence is above average. We suggest that a tendency to overrate one’s cognitive abilities may be a stable feature of human psychology.

## Introduction

The statement that a majority of people claim to be more intelligent than average is literally a textbook example of overconfidence and self-enhancement [1–6]. Here we ask whether such “intelligence overconfidence” is reliably found in large samples weighted to be nationally representative, differs by method of data collection (telephone or online), and varies according to demographic factors including sex, age, and race/ethnicity. The answers to these questions will help solidify the evidence base for popular claims in psychology and contribute to research on self-perceptions, overconfidence, and intelligence.

Most demonstrations of the “smarter than average” effect are conducted using convenience samples, a method that raises concerns about generalizability [7,8]. Some studies have improved upon convenience sampling by collecting nationally representative survey data from college [9] and high school [10] students to measure change in self-positivity and narcissism over time. However, student populations suffer the limitations of failing to represent older and less-educated people, differing from the general population in income, race/ethnicity, and sex, and potentially having difficulty imagining the “average person” outside of a university environment.

Sampling from a more representative source of participants can overcome these limitations. Applying probability weighting to the sample can then account for over- and under-sampling of demographic groups. Some representative surveys of people’s beliefs about their own intelligence have been reported in the media [11,12]. However, these reports do not include important methodological details like sample sizes, weighting schemes, and inferential statistics. The only published study of a nationally representative sample of Americans reporting overconfident beliefs about relative intelligence was conducted over 50 years ago [13]. For these reasons, we decided to examine the pattern of intelligence overconfidence in the present U.S. population. From two large samples weighted to be nationally representative, drawn using distinct polling methods (telephone and online), with the second constituting a replication of the first, we report the proportions of Americans who agreed with the statement, “I am more intelligent than the average person”.

Although self-enhancement and overconfidence have been demonstrated across a broad range of traits [14,15], we chose to focus on the specific trait of intelligence because of its practical and theoretical importance. Because intelligence is normally distributed (when measured as IQ), rather than skewed like many other desirable traits [14], 50% of people in the general population will be above average (i.e., the mean and the median are the same). Additionally, general intelligence is consistently and readily measured [16], predictive of a wide variety of positive outcomes [17], relevant to trait-level overconfidence [18], and broadly perceived as highly desirable [1]. Measuring population-representative beliefs about this trait allows us to draw specific conclusions about possible demographic differences that are exploratory (e.g., sex, age, and race/ethnicity) [19] and theoretically informed (e.g., education level). With the results of each survey weighted to United States Census population data, we can directly compare patterns across survey methods (e.g., telephone and online) and demographic categories. Moreover, our approach updates the only similar study, conducted over 50 years ago [13], and improves upon it by examining whether, as a population, Americans have a calibrated sense of their own intelligence [20,21]. Specifically, we asked whether college-educated respondents, who on average *are* more intelligent than the average person, correctly *believe* that they are more intelligent than average.

## Method

### Survey methods

#### Telephone

A large telephone survey (*N* = 1,838) was conducted in June of 2009 by the polling company SurveyUSA using random digit-dialing to contact land-line telephone users in the United States. Volunteer participants answered a series of questions read by a pre-recorded female voice program by using their telephone’s keypad. Approximately 2.3% of the 79,014 random digit calls yielded a complete response. This response rate is typical of automated calling surveys and is associated with acceptably low amounts of sampling bias [22]. All data were collected anonymously.

#### Online

A large sample of respondents (*N* = 983) was recruited via Amazon Mechanical Turk (MTurk) in July-August of 2011. The listing advertised that respondents would complete a “short survey of your beliefs about psychology.” Each participant was paid $0.25. Recruitment was restricted to the United States and repeat IP addresses were blocked from taking the survey. Questions were presented in the same order as the telephone survey, but appeared on screen instead of being read aloud. Of 1,020 people recruited, only 37 did not complete the survey.

These sample sizes exceeded the minimum required to detect a significant difference from 50% agreement (which would indicate no overconfidence at the population level) with 99% power. Our studies were not preregistered because they were conducted before preregistration was common in psychology. Therefore, all statistical inferences we draw may be regarded as exploratory rather than confirmatory.

### Procedure

The telephone survey originally was designed to achieve a nominal, nationally representative sample of 1,500 participants after weighting to the 2000 U.S. Census, and the online survey was designed to achieve a nominal sample of 750 after weighting to the 2010 census. The telephone survey was re-weighted to achieve a nominal sample of 750 participants based on 2010 census figures to allow for a direct comparison between the surveys. These sizes are typical of representative public opinion and political polls. In each case, we recruited more than the nominal number of participants to ensure adequate representativeness of the U.S. population after weighting. In addition to the item “I am more intelligent than the average person,” participants responded to items regarding popular myths about memory, attention, and the brain (full survey available at https://osf.io/zkh3e/?view_only=57b247e35eb4496399f40ca20cdf635f). These results are reported elsewhere [23–26].

For each item, participants chose one of five possible responses (Strongly Agree; Mostly Agree; Mostly Disagree; Strongly Disagree; Don’t Know). Additionally, participants provided the following information: sex, age, race/ethnicity, education, household income, region, number of psychology classes ever taken, and number of psychology books read in the last three years. All participants answered the same questions in the same order, and response options were always presented in the order shown above. All research reported in this manuscript was conducted with approval from the IRB of the University of Illinois.

### Weighting to U.S. Census

To directly compare both surveys, we weighted each to a nominal nationally representative sample of 750 nationally Americans using the 2010 U.S. Census demographics for sex (male, female), age (< 44, ≥ 44; based on median age), and race/ethnicity (white, nonwhite). Dichotomous weighting accounts for the over- or under-sampling for each combination of demographics in each polling method and is standard practice in polling and survey methodology. A greater proportion of women and older Americans completed the telephone survey and a greater proportion of younger Americans completed the online survey. Table 1 displays the raw sample sizes and demographic weightings applied to each sample. All data are publicly available (https://osf.io/zkh3e/?view_only=57b247e35eb4496399f40ca20cdf635f).

**Table 1:** Weighted sample proportions and demographic weighting values obtained from 2010 U.S. Census data.

Sex	Age	Race / Ethnicity	Raw Telephone *N*	Raw Online *N*	Proportion of weighted population	Telephone weights for *N* = 750	Online weights for *N* = 750
Female	Young	Nonwhite	47	116	0.076	1.213	0.491
		White	114	312	0.181	1.192	0.435
	Old	Nonwhite	112	14	0.076	0.509	4.072
		White	878	135	0.181	0.155	1.006
Male	Young	Nonwhite	27	81	0.072	1.994	0.665
		White	93	254	0.171	1.380	0.505
	Old	Nonwhite	89	8	0.072	0.605	6.729
		White	478	63	0.171	0.268	2.037

## Results

**Fig 1:**
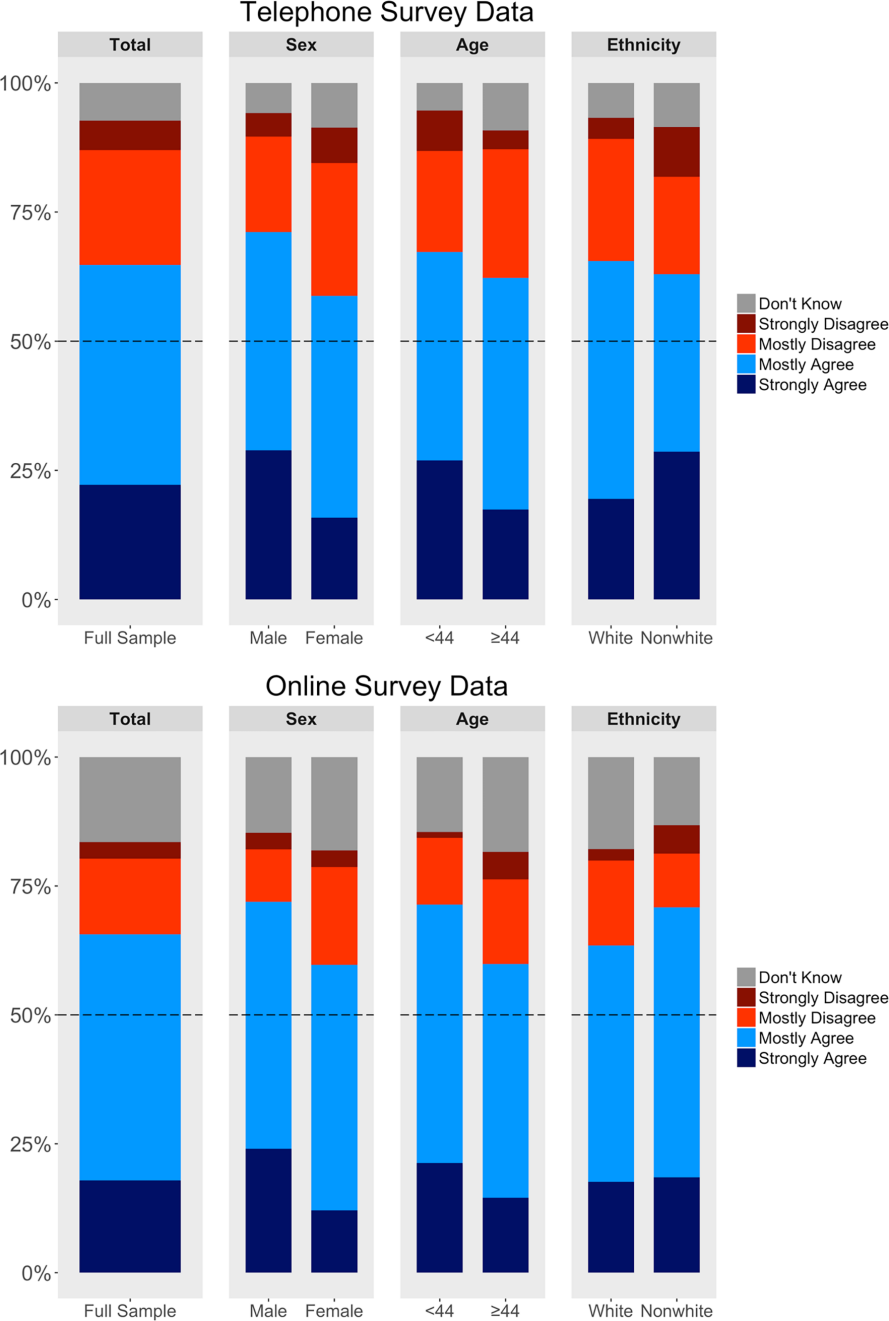
Percentages of participants reporting each level of agreement with the statement, “I am more intelligent than the average person”. Weighted to 2010 U.S. Census categories for sex, age, and race/ethnicity. Top: telephone survey; Bottom: online survey.

Combining both surveys (resulting in 1,500 total weighted participants), 65% of participants agreed with the statement “I am more intelligent than the average person,” with 20% choosing “Strongly Agree” and 45% choosing “Mostly Agree.” The remaining 35% of participants included those who chose “Mostly Disagree,” “Strongly Disagree,” or “Don’t Know.” Because we classified “Don’t Know” responses as not agreeing, 65% represents a conservative estimate of the proportion of people who place themselves above average. Considering only people who expressed an opinion (i.e., excluding all “Don’t Know” responses), nearly three times as many people agreed (65%) as disagreed (23%) that they are above average in intelligence. Fig 1 presents, for each weighted demographic category, the percentage of participants choosing each level of agreement (Tables 2–5 display results for all demographic categories). Here and for subsequent results, we include two-tailed *p*-values for their heuristic value, but they should be treated as the outcomes of exploratory analyses rather than as confirmatory tests [27]. We report group differences using the *z*-test for differences between two independent proportions along with the 95% confidence interval around the difference.

**Table 2:** Demographic report of weighted survey data (telephone).

Weighted Categories	Demographic Category	Strongly Agree	Mostly Agree	Mostly Disagree	Strongly Disagree	Don’t Know	*n*
Sex	Male	29%	42%	18%	5%	6%	364
	Female	16%	43%	26%	7%	9%	386
Age	< 44 years	27%	40%	20%	8%	5%	375
	≥ 44 years	17%	45%	25%	4%	9%	375
Race	White	19%	46%	24%	4%	7%	528
	Nonwhite	29%	34%	19%	10%	9%	222
Total	All	22%	43%	22%	6%	7%	750

*Note*. Proportions of responses to the statement, “I am more intelligent than the average person,” collected via telephone survey, weighted for Sex, Age, and Race/Ethnicity.

**Table 3:** Demographic report of unweighted categories (telephone).

Unweighted Categories	Demographic Category	Strongly Agree	Mostly Agree	Mostly Disagree	Strongly Disagree	Don’t Know	*n*
Income	< 40K /year	14%	40%	30%	6%	11%	728
	40K–80K /year	14%	51%	25%	3%	8%	622
	> 80K /year	25%	47%	19%	3%	5%	393
Region	South	17%	44%	27%	3%	8%	693
	Northeast	17%	47%	21%	4%	11%	344
	West	17%	46%	24%	5%	8%	283
	Midwest	15%	44%	27%	4%	9%	518
Education	No College	12%	38%	36%	5%	9%	480
	Some College	14%	45%	26%	5%	10%	671
	Graduated College	20%	53%	18%	1%	7%	300
	Graduate Degree	24%	48%	17%	3%	7%	372
Total	All	17%	45%	25%	4%	9%	1838

*Note*. Proportions of responses to the statement, “I am more intelligent than the average person,” collected via telephone survey. All categories are unweighted. Participants who did not specify their income (*n* = 95) or education level (*n* = 15) were excluded from analysis within these categories.

**Table 4:** Demographic report of weighted survey data (online).

Weighted Categories	Demographic Category	Strongly Agree	Mostly Agree	Mostly Disagree	Strongly Disagree	Don’t Know	*n*
Sex	Male	24%	48%	10%	3%	15%	364
	Female	12%	48%	19%	3%	18%	386
Age	< 44 years	21%	50%	13%	1%	15%	375
	≥ 44 years	15%	45%	16%	5%	18%	375
Race	White	18%	46%	16%	2%	18%	528
	Nonwhite	19%	52%	10%	5%	13%	222
Total	All	18%	48%	15%	3%	16%	750

*Note*. Proportions of responses to the statement, “I am more intelligent than the average person,” collected via online survey, weighted for Sex, Age, and Race/Ethnicity.

**Table 5:** Demographic report of unweighted categories (online).

Unweighted Categories	Demographic Category	Strongly Agree	Mostly Agree	Mostly Disagree	Strongly Disagree	Don’t Know	*n*
Income	< 40K /year	19%	47%	16%	2%	16%	502
	40K–80K /year	17%	48%	16%	2%	18%	318
	> 80K /year	21%	56%	9%	0%	13%	163
Region	South	18%	49%	14%	3%	17%	358
	Northeast	22%	49%	15%	3%	12%	193
	West	21%	53%	14%	1%	11%	212
	Midwest	16%	45%	17%	1%	22%	220
Education	No College	20%	39%	21%	3%	17%	121
	Some College	18%	48%	15%	2%	17%	453
	Graduated College	17%	51%	13%	2%	17%	241
	Graduate Degree	22%	54%	13%	0%	11%	167
Total	All	19%	49%	15%	2%	16%	983

*Note*. Proportions of responses to the statement, “I am more intelligent than the average person,” collected via online survey. All categories are unweighted. One participant did not specify level of education and was excluded from analysis within that category.

### Comparing telephone and online surveys

Before weighting to the U.S. census, a greater percentage of online survey participants (68%) than telephone survey participants (62%) claimed to be more intelligent than average (difference = 5.9%, 95% CI: [2.2%, 9.6%]), *z* = 3.11, *p* = .002. This difference diminished after weighting (telephone: 65% agreement; online: 66% agreement, difference = 0.9%, 95% CI: [–3.9%, 5.7%]), *z* = .33, *p* = .75, indicating that overall agreement did not differ between weighted samples.

### The smarter than average effect within weighted demographic categories

*Sex*. In both surveys, men were more likely to agree that they are more intelligent than average than were women (telephone: 71% vs. 59%, difference = 12.4%, 95% CI: [5.5%, 19.0%]), *z* = 3.54, *p* < .001; (online: 72% vs. 60%, difference = 12.2%, 95% CI: [5.6%, 19.0%]), *z* = 3.57, *p* < .001.

Men were much more likely to *“*strongly agree” with the intelligence statement than were women (telephone: 29% vs. 16%, difference = 13.0%, 95% CI: [7.1%, 18.9%]), *z* = 4.30, *p* < .001; (online: 24% vs. 12%, difference = 11.9%, 95% CI: [6.5%, 17.5%]), *z* = 4.28, *p* < .001. Men and women chose “mostly agree” in similar proportions (telephone: 42% vs. 43%, difference = 0.7%, 95% CI: [–6.4%, 7.7%]), *z* = .19, *p* = .85; (online: 48% vs. 48%, difference = 0.3%, 95% CI: [–7.0%, 7.3%]), *z* = .04, *p* = .97. Thus, the overall sex difference was driven by differences in participants who “strongly agree” that they are more intelligent than the average person.

*Age*. Younger Americans (< 44 years) were more likely to agree than were older Americans (≥ 44 years) in the online survey (71% vs. 60%, difference = 11.6%, 95% CI: [4.9%, 18.4%], *z* = 3.38, *p* < .001), but this difference was smaller in the telephone survey (67% vs. 62%, difference = 5.0%, 95% CI: [–1.2%, 11.8%], *z* = 1.45, *p* = .147). To ensure that the age effect observed in each survey was not a spurious result of dichotomization, we regressed the dichotomized agreement variable on the continuous unweighted measure of age using logistic regression. In both surveys, younger Americans were more likely to agree: telephone exp(B) = .992, 95% CI: [.986, .997], Wald = 7.96, *p* = .005; online exp(B) = .978, 95% CI: [.969, .989], Wald = 17.37, *p* < .001.

Younger Americans (< 44 years) were more likely to “strongly agree” than were older Americans (≥ 44 years) (telephone: 27% vs. 17%, difference = 9.5%, 95% CI: [3.7%, 15.5%]), *z* = 3.17, *p* = .002; (online: 21% vs. 15%, difference = 6.7%, 95% CI: [1.5%, 12.4%]), *z* = 2.48, *p* = .013. Younger and older adults were comparably likely to respond “mostly agree” (telephone: 40% vs. 45%, difference = 4.5%, 95% CI: [–2.5%, 11.5%]), *z* = 1.30, *p* = .21; (online: 50% vs. 45%, difference = 4.8%, 95% CI: [–2.3%, 11.9%]), *z* = 1.32, *p* = .19.

*Race/ethnicity*. White and nonwhite Americans showed similar tendencies to believe that they were smarter than average in the telephone survey (66% vs. 63%, difference = 2.5%, 95% CI: [–4.9%, 10.1%], *z* = .646, *p* = .518) and online survey (64% vs. 71%, difference = 7.4%, 95% CI: [–0.2%, 14.3%], *z* = 1.91, *p* = .056).

Non-white Americans were more likely to “strongly agree” than were white Americans on the telephone survey (29% vs. 19%, difference = 9.2%, 95% CI: [2.7%, 16.4%]), *z* = 2.80, *p* = .005, but not on the online survey (19% vs. 18%, difference = 0.9%, 95% CI: [–4.9%, 7.2%]), *z* = .28, *p* = .78. White participants were more likely than non-white participants to select “mostly agree” on the telephone poll (46% vs. 34%, difference = 11.7%, 95% CI: [4.1%, 19.1%]), *z* = 2.98, *p* = .001, but this pattern was smaller and in the opposite direction in the online poll (white: 46% vs. nonwhite: 52%, difference = 6.5%, 95% CI: [–1.4%, 14.1%]), *z* = 1.61, *p* = .11.

### Education: Are beliefs calibrated?

**Fig 2:**
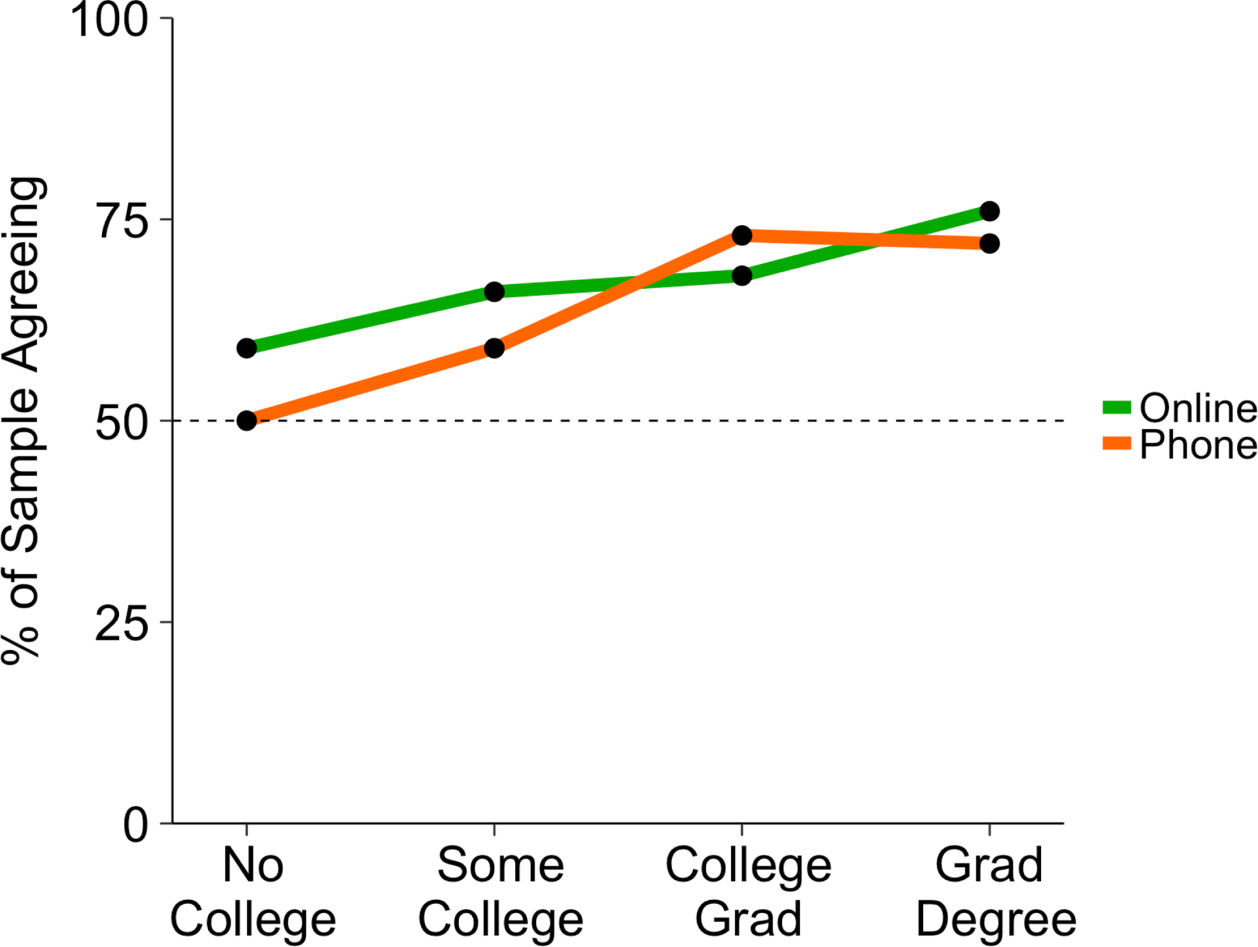
Percentages of participants agreeing with the statement, “I am more intelligent than the average person,” grouped by education level. Agreement was measured as selecting either “Strongly Agree” or “Mostly Agree”.

In both surveys, people with more education were more likely to claim above-average intelligence (see Fig 2). A linear contrast of education level, assigning contrast weights of –3, –1, 1, and 3 to the education levels of no college, some college, college graduate, and graduate degree, predicted unweighted agreement (disagreement and agreement were assigned 0 and 1, respectively) in both the telephone survey (contrast estimate = 0.802, 95% CI: [0.597, 1.01], *t*(1819) = 7.87, *p* < .001) and the online survey (contrast estimate = 0.564, 95% CI: [0.228, .900], *t*(978) = 3.29, *p* = .001). Although the measured education levels are not linear *per se*, the typical number of years of education required to attain each level follow a nearly linear structure. The proportion of Americans in our samples holding a college degree or higher (telephone: 36%; online: 42%) approximated the 2010 U.S. Census figure of 36%. Consequently, our “smarter than average” effect across education levels is unlikely to have resulted from oversampling highly-educated people.

What proportions of people *should* claim above-average intelligence? Given that the average college graduate has an IQ approximately 13–15 points (one standard deviation) above the population mean (based on WAIS norms [28] and Bureau of Labor Statistics NLSY-97 data [29]), we compared the proportion of participants with a college or graduate degree who agreed that they were more intelligent than average (telephone: 73%; online: 73%) with the proportion of college graduates who could be considered more intelligent than the average American (84% by one account [28], or 81% by another [29]). This result suggests that college graduates in our samples actually slightly *underestimated* their relative intelligence. Conversely, data in the NLSY-97 study put the average IQ score for Americans with a high school diploma or GED at 99, which implies that only 47% of individuals in this category can be considered above average [29]. Of those in our sample who reported “no college” or “some college,” 55% of the telephone sample and 62% of the online sample claimed above-average intelligence. This result suggests that relatively uneducated participants tended to *overestimate* their relative intelligence [30,31]. Because only a minority of Americans have college degrees, members of the population as a whole tended to somewhat overestimate their relative intelligence.

## Discussion

Two surveys, weighted to be nationally representative (total *N* = 2,821), found that nearly two-thirds of Americans believe that they are more intelligent than average. The survey methods (telephone, online) yielded similar overall agreement rates after weighting responses to match the U.S. population in sex, age, and race/ethnicity. In both surveys, men were more likely to express confidence in their intelligence than were women, and younger people were somewhat more likely to agree with the claim than older people.

These beliefs about relative intelligence appeared to be somewhat calibrated: Highly educated individuals were more likely to agree that they are more intelligent than the average person, whereas relatively uneducated individuals were less likely to agree [21, 31, 32]. Still, even among the least educated group of respondents, 50% or more agreed that they were above average in intelligence. These findings are consistent with several major theories of overconfidence: that the least intelligent are the most overconfident [30]; that self-perceptions are somewhat calibrated to reality [33]; and that comparative self-judgments regress toward the mean when collected from groups of educated and uneducated individuals [34, 35].

Our results do not explain *why* 65% of Americans agree that they are more intelligent than average. Several explanations are plausible [36]. First, although one-item, self-report measures of global intelligence correlate positively with IQ scores [37], participants may conceive of intelligence more broadly [38] and select that aspect of intelligence where they believe they outperform others. If so, more than 50% of people might actually be above average in some aspect of intelligence even if only 50% can be above average on IQ. Still, our finding that more educated people are more likely to agree suggests that participants are thinking to at least some extent about general intelligence.

Second, people may choose different baselines when comparing themselves to “the average person.” If people define “average” differently, perhaps based on who they encounter regularly [39], then more than 50% of respondents might report greater than average intelligence. Note that for this possibility to hold true and to be inconsistent with overly optimistic beliefs, people would need to systematically calibrate their notion of average downward (less intelligent people would need to choose a lower “average” than more intelligent people). Finally, it may simply be the case that people are somewhat calibrated, though overly optimistic on average, in their beliefs about their own intelligence [35].

Because these results were collected from and weighted based on the United States population, we caution against generalizing our findings before they are replicated in other cultures and regions. Our methodology was limited by the static question order presented to participants. Although we had no *a priori* reason to expect an order effect in this context, future research should consider this possibility. In a nationally representative study of Americans’ beliefs about competency in handling firearms, overconfidence was measured using a similar one-item, direct comparison measure [40]. The authors reported no difference in overconfidence regardless of whether or not there was a neutral scale midpoint. These results were similar across 2-, 3-, 5-, and 13-point rating scales. Thus, we have no reason to believe that including a neutral midpoint would have meaningfully affected our results. The education-based analysis was limited to comparisons based on population characteristics, not objectively measured individual performance.

Despite these limitations, we conclude that Americans’ self-flattering beliefs about intelligence are alive and well several decades after their discovery was first reported. Our results update the textbook phenomenon of intelligence overconfidence by (1) replicating the effect using large, representative, contemporary samples and two distinct survey methods, (2) demonstrating a degree of calibration across levels of education, and (3) showing moderation based on sex and age. The endurance of the smarter-than-average effect is consistent with the possibility that a tendency to overrate one’s own abilities is a stable feature of human psychology.
